# Effects of concurrent HER2-directed therapy on development of cerebral radionecrosis after stereotactic radiotherapy: a systematic review

**DOI:** 10.1007/s00066-025-02416-w

**Published:** 2025-06-10

**Authors:** Clara Grinzinger, Georg Stüben, Maria Neu, Anna Rubeck, Stefan Schiele, Lars Behrens, Klaus-Henning Kahl

**Affiliations:** 1 Klinik für Strahlentherapie und Radioonkologie, Universitätsklinikum Augsburg, Stenglinstraße 2, 86156 Augsburg, Germany; 2 University of Augsburg, Institute of Mathematics, Universitätsstraße 2, 86159 Augsburg, Germany; 3 Klinik für Diagnostische und Interventionelle Neuroradiologie, Universitätsklinikum Augsburg, Stenglinstraße 2, 86156 Augsburg, Germany

**Keywords:** Brain metastases, Radionecrosis, Breast cancer, Targeted therapies, Trastuzumab

## Abstract

**Purpose:**

With increasing use of human epithelial growth factor receptor two (HER2)-targeted therapies, outcomes for numerous breast cancer patients have improved. Nevertheless, patients with HER2-positive tumours face a comparatively heightened risk for developing brain metastases (BM), which are often treated with stereotactic radiosurgery (SRS). Radionecrosis represents one of the clinically most significant adverse events of SRS. However, a knowledge gap remains regarding the effects of concurrent use of HER2-targeted therapies with SRS on development of radionecrosis, given conflicting findings in existing studies.

**Methods:**

This systematic review was conducted in May 2024 through a search across electronic databases PubMed/MEDLINE and Cochrane library and was supplemented by citation searching and an artificial intelligence (AI) search.

**Results:**

The literature search yielded 194 articles. After applying eligibility criteria, a total of 13 studies with 3219 patients total were included, with approximately 270 patients in the topic-relevant subgroup. Investigated substances vary in different publications and include HER2 antibodies, antibody–drug conjugates (ADCs), such as trastuzumab emtansine (T-DM1), and kinase inhibitors. Four of six studies on ADCs demonstrated a higher risk for radionecrosis with concurrent administration. Two studies on lapatinib found no significant effects, as did as most studies investigating mainly HER2 antibodies. One publication reported an even lower risk for radionecrosis (RN) with concurrent use of HER2/EGFR tyrosine kinase inhibitors (TKIs).

**Conclusion:**

While concurrent use of T‑DM1/ADCs seems associated to elevated radionecrosis risk, an ambiguous situation for other substances persists. Heterogenous study designs with varying substances, definitions of concurrent use, and radionecrosis parameters must be considered. Included studies are partly limited by sample size and retrospective study design. Therefore, clinical implications remain difficult to claim; further research on this topic is needed.

## Introduction

Breast cancer poses a significant global health challenge, affecting millions of patients each year. Annually there are approximately 70,000 new cases in Germany, making breast cancer the most widespread cancer among women [1]. Within its subtypes, human epidermal growth factor receptor 2 (HER2)-positive breast cancer represents a special entity, historically characterized by being more aggressive and having a poorer prognosis. However, in recent years, there is a rising importance and growing use of targeted therapies. HER2-monoclonal antibodies like trastuzumab and pertuzumab, small molecule drugs/kinase inhibitors like lapatinib as well as antibody–drug conjugates (ADC), like trastuzumab emtansine (T-DM1), have significantly improved outcomes and overall survival for this patient subgroup [2].

Brain metastases represent a dreaded complication in the course of disease, affecting approximately a quarter of patients with advanced breast cancer [3, 4]. The risk for developing brain metastases in HER2-positive patients seems to be higher than in other subtypes [5–7]. Management options for brain metastases mainly include neurosurgical resection, systemic therapy, and radiation therapy. As patients are surviving increasingly longer, therapy-related late toxicity is becoming more notable. Hence, whole brain irradiation (WBI) is being used more restrictively and most patients are treated with stereotactic radiosurgery (SRS) [8–10]. One of the most important and clinically significant adverse outcomes of SRS is radionecrosis. Known risk factors for radionecrosis are large treated volumes, prior whole brain irradiation and higher prescription dose [2, 11, 12].

Despite advances in the oncological therapy of patients with HER2-positive breast cancer brain metastases (BCBM), a knowledge gap persists. Regardless of its efficacy, there remains uncertainty regarding the systemic treatments adverse events when administered concurrently with stereotactic radiation of brain metastases. This is especially the case for a potential higher risk of radionecrosis, with only little research performed on this specific topic and different studies showing partly conflicting results [13–15].

Moreover, for targeted therapies such as BRAF/MEK inhibitors, an association with increased risk for radionecrosis when administered simultaneously has been shown [16].

Furthermore, diagnosis of radiation necrosis can be difficult to achieve. There is no sole method of diagnostic imaging with a specificity high enough to differentiate between local recurrence and radiation necrosis safely and routinely. Indicative options for diagnostic imaging include magnetic resonance imaging (MRI), perfusion MRI and 18F-fluorethylthyrosine-positron emission tomography (FET-PET), but histology remains the gold standard for definite diagnosis [17–19].

Additionally, pathomechanisms underlying the development of cerebral radionecrosis remain only partly understood, although a dysregulation of aquaporin 4 channels has been demonstrated by Stumpf et al. [20]. Other attempts at explanation include an increased secretion of hypoxia-inducible factor-1-alpha (HIF-1-α) due to vascular damage, leading to increased vascular endothelial growth factor (VEGF) [19].

This review will systematically examine the existing literature to answer the question, what effect the concurrent use of HER2-targeted therapies has on developing radiation necrosis after stereotactic radiation of brain metastases. By giving an overview about existing studies on this topic, we hope to provide insights into optimizing therapeutic strategies for patients with HER2-positive breast cancer brain metastases (BCBM) and show implications for future clinical use and directions for research on radionecrosis and HER2-targeted therapies.

## Materials and methods

This literature review was performed in May 2024 to identify clinical studies concerning risk of radionecrosis in concurrent use of HER2-targeted therapies with stereotactic radiation of brain metastases.

### Definitions

In this context, it is fundamental to accurately define the used terminology. A recent guideline published by the German Society of Radiation Oncology (DEGRO) proposed a distinct nomenclature. Due to prolonged survival and changes in oncological therapies, long-term side effects are increasing. In terms of cerebral radiosurgery, these often appear as contrast-enhancing lesions (CEL) on imaging. Although challenging, it is important to differentiate between the different causes of CEL, as clinical presentations, prognosis and need of treatment varies. Predominantly, CEL can occur as radiation-induced blood–brain barrier disruption (BBD), also called “pseudo progression”. BBD typically occurs earlier and both within and out of field of the high-dose radiation volume. Usually, symptoms are only mild and often self-limiting or reversible and oedema is only small [19].

This has to be differentiated from radiation necrosis (RN), which is irreversible radiation-induced brain tissue damage. RN has a clearer dose–volume dependency and usually manifests in the high-dose-treated region. Due to differences in the pathomechanism, oedema in RN is usually more pronounced, causing more and potentially irreversible, life-threatening symptoms. That said, RN can also present in an asymptomatic way, but often has a more progressive and rapid progression pattern. As RN mostly requires treatment when symptomatic, some clinical studies opt to employ clinically significant RN (CSRN) or symptomatic RN (SRN) as outcome parameters. RN typically occurs later (within 6–18 months after RT). However, RN can also occur shortly after RT as “early RN”, and after several years as “late/ultra-late RN”. These differences in timing as well as possible occurrence of mixed forms (especially after higher-dose RT) and transitions between both can possibly lead to misdiagnosis and mistreatment as BBD in the first few months or as tumour progression after several years [19].

### Search strategy

The search was conducted across electronic databases PubMed/MEDLINE and Cochrane library. Additionally relevant studies were identified through citation searching and searches using common artificial intelligence research tools, including for example scite.ai and consensus.ai. The search strategy in electronic databases consisted of terms and keywords related to HER2-targeted therapies, such as “HER2”, “HER-2”, “trastuzumab”, “pertuzumab”, “lapatinib”, “neratinib”, “tucatinib”, and radionecrosis, such as “radionecrosis” or “radiation necrosis” in combination with medical subject headings (MeSH) terms (in particular “radiosurgery/adverse effects” or “radiosurgery”). The full search strategy is available upon request. The literature search and selection process were performed adhering to the Preferred Reporting Items for Systematic Reviews and Meta-Analyses (PRISMA) guidelines whenever possible.

### Study selection (inclusive inclusion and exclusion criteria)

Titles and abstracts of search results were screened for relevance to the subject matter, integrated into the EndNote 21 citation software, and duplicates were removed. Articles were retrieved and tested for eligibility based on the following inclusion criteria: clinical study, stereotactic radiation, existence of brain metastases, minimum of 5 lesions included in the study, HER2 mutation, use of HER2 targeted therapy, English or German language, published in 2010 or later. Papers published as case reports, case series, reviews, metanalyses, or articles were excluded, as well as publications not investigating radionecrosis as an endpoint and studies focusing on other radiation techniques like whole brain irradiation. Due to practical and organizational reasons the process of search, screening and eligibility testing was only performed by one reviewer.

### Data extraction

Data extraction was also performed by one reviewer due to practical and organizational reasons. Extracted data included study characteristics (author, year of publication, study design), number of participants, definition of concurrent use, exact substance, radiation characteristics (dose, fractions), type of outcomes regarding radionecrosis, approach to diagnostic confirmation, statistical methods, median follow-up and results relevant to the research question. Missing information on collected variables were requested from the corresponding authors.

### Data synthesis

The results of included studies were synthesized narratively to account for expected heterogeneity among different studies.

## Results

### Summary of findings

**Fig. 1:**
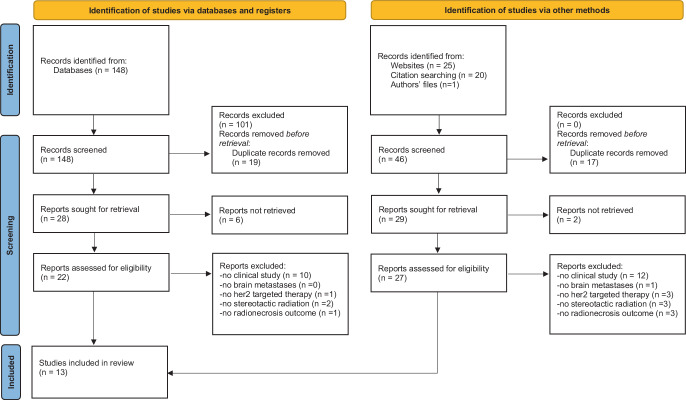
Study selection process: Modified Preferred Reporting Items for Systematic Reviews and Meta-Analyses (PRISMA) 2020 flow diagram for new systematic reviews which included searches of databases, registers and other sources

Our review identified a total of 194 search results through the above mentioned search strategies, from which 93 were categorized as generally relevant to the research topic by the reviewer. Subsequently, 36 duplicates were eliminated, resulting in 57 references, of which 49 could be successfully retrieved and evaluated for eligibility. Within this group, the following 13 studies met all predefined criteria and were therefore included in this review: [13, 14, 20–30]. A total of 36 titles did not meet the inclusion criteria due to nonsuitable publication type (*n* = 22), not examining brain metastases (*n* = 1), not analysing HER2-targeted therapies (*n* = 4), not evaluating stereotactic radiation in particular (*n* = 5) or not providing outcomes on cerebral radionecrosis (*n* = 4). The selection process is shown in Fig. 1.

Included studies were published between 2016 and 2024 and collectively represent a total of 3219 participants, with approximately 270 patients falling within topic-relevant subgroups. All included studies were designed as retrospective analyses. Within the 13 included studies, six investigated the administration of T‑DM1 or other ADCs [20–22, 25, 26, 30], while two studies examined lapatinib [14, 24] and one publication focused on tucatinib [27]. Four studies included multiple HER2− directed therapies (like trastuzumab) [13, 23, 28, 29]. Concurrent administration was defined as occurring within a 6 month window [22], within a 1 month/30 day/4 week window [20, 25, 28–30], within a 21 day window [23, 26] and within five biological half-lives [13, 14, 24]. Additionally, outcome parameters regarding radionecrosis showed substantial variation, with ten utilizing general occurrence of radionecrosis over time [13, 14, 21–25, 27, 28] or 12 months post radiation [29] and three employing clinically significant radionecrosis (CSRN) [20] or symptomatic radionecrosis (SRN) [26, 30] as endpoints. Some additionally covered symptoms and hospital admissions. Most publications followed similar multidisciplinary methods and processes for diagnosing radionecrosis. The statistical analysis was predominantly performed using competing risk regression analysis, cumulative incidence analysis, logistic regression, and Gray’s test. However, three studies opted to only describe events of radionecrosis narratively or with descriptive statistics due to small sample sizes [22, 23, 25], while one study solely used descriptive statistics and Pearson’s Chi-squared test because of a fixed-time outcome measure [29]. One publication did not provide any information regarding statistical analysis of radionecrosis [27]. A summary of relevant study characteristics and main findings can be found in Tables 1 and 2.

**Table 1:** Summary of included studies

Authors/ year/ study design	Total sample size (patients)	Subgroup sample size^a^	Substances	Age (years)^b^	Definition of “concurrent”	Outcome parameters regarding RN	Median follow-up (months)	Results
Id Said et al./ 2022/ R[21]	67	Max. 29 ^c^	T‑DM1	Mean 53.6	Only pre/post SRS	RN	15.6	Higher risk for T‑DM1 after SRS
Mills et al./ 2021/ R [22]	16	19 lesions	T‑DM1	56	6‑month window	RN	13.2	One case of RN, no statistical analyses
Stumpf et al./ 2019/ R [20]	45	16	T‑DM1	45	4‑week window	CSRN	Not stated	Higher risk for concurrent T‑DM1
Ippolito et al./ 2022/ R [23]	49	10	Trastuzumab + pertuzumab	57	21-day window	RN	18.3	One case of RN, no statistical analyses
Miller et al./ 2017/ R [13]	547	193 lesions	HER2-antibodies and HER2/EGFR TKI	54	5 biological half-lives	RN	10.1	Lower risk for concurrent HER2/EGFR TKI
Kim et al./ 2019/ R [24]	84	18	Lapatinib	52	5 biological half-lives	RN	8.2	No significant effect
Geraud et al./ 2017/ R [25]	12	4	T‑DM1	Mean 46	1‑month window	RN	Not stated	Higher risk for concurrent T‑DM1
Lebow et al./ 2023/ R [26]	98	35	T‑DM1, TDXd	55	7 days before / 21 days after SRS	SRN	12.4	Higher risk for concurrent ADCs
Khatri et al./ 2023/ R [27]	22	17 treatment sessions	Tucatinib	50.7	Not stated	RN	20.8^d^	No analyses for concurrent versus sequential
Parsai et al./ 2019/ R [14]	126	24	Lapatinib	Mean 54	5 biological half-lives	RN	17.1	No significant effect
Miller et al./ 2016/ R [28]	1939	47	HER2 antibodies and lapatinib	58 (RN group); 60 (control)	30-day window	RN	12^e^	No significant effect
Park et al./ 2022/ R [29]	46	24	HER2-directed therapy	Mean 53.3 (RN), 50.4 (control)	4‑week window	RN 12 months post-SRS	12	No significant effect for any substance but for higher number of administered drugs
Koide et al./ 2024/ R [30]	168	19	T‑DM1, TDXd	57	4‑week window	SRN	31	Higher risk for concurrent ADCs

*R* retrospective study design, *T‑DM1* trastuzumab emtansine, *TDXd* trastuzumab deruxtecan, *RN* radionecrosis, *CSRN* clinically significant radiation necrosis, *SRN* symptomatic radiation necrosis, *ADCs* antibody-drug conjugates

^a^ patients if not otherwise stated

^b^ median ages if not otherwise stated

^c^ no exact value available

^d^ from tucatinib initiation

^e^ longer for lesions developing RN

**Table 2:** Radiotherapy characteristics (prescription dose and fractionization)

Author/year	SRS (rate and characteristics)	FSRT (rate and characteristics)	WBI (rate and characteristics)
Id Said et al./ 2022 [21]	78% (15–24 Gy/1 fraction)	22% (24–32 Gy/3–5 fractions)	–
Mills et al./ 2021 [22]	60% (median dose 21 Gy (14–24 Gy)/ 1 fraction)	40% (median dose 25 Gy (20–30)/ median fractions 5 (3–5))	–
Stumpf et al./ 2019 [20]	For RN lesions: median dose 20 Gy (18–25), median fractions 1 (1–5)		–
Ippolito et al./ 2022 [23]	–	100% (median dose 27 Gy (12–27)/ median dose per fraction 9 Gy (4–9))	–
Miller et al./ 2017 [13]	100% (24 Gy (IQR 18–24))	–	–
Kim et al./ 2019 [24]	100% (median dose 24 Gy (18–24))	–	–
Geraud et al./ 2017 [25]	100% No details available	–	–
Lebow et al./ 2023 [26]	73.85% (59% 21 Gy/1 fraction, 13.8% 18 Gy/1 fraction, 1.05% other)	26.05% (16.3% 27 Gy/3 fractions, 8.7% 30 Gy/5 fractions, 1.05% other)	–
Khatri et al./ 2023 [27]	72% (median dose 24 Gy (16–24))	28% (median dose 27 Gy (20–50))	–
Parsai et al./ 2019 [14]	Dose according to RTOG 90–05 protocol: 18 Gy for tumours < / = 20 mm, 15 Gy for 21–30 mm; 12 Gy for 31–40 mm		–
Miller et al./ 2016 [28]	100% (median dose 24 Gy (18–24))	–	–
Park et al./ 2022 [29]	60.9% (1 fraction) (total median dose 20 Gy) (total mean dose 21.9 Gy)	39.1% (5 fractions) (total median dose 20 Gy) (total mean dose 21.9 Gy)	–
Koide et al./ 2024 [30]	27.4% (18–22 Gy in 1 fraction)	32.1% (17.3% 27–30 Gy in 3 fractions; 12.5% 30–35 Gy in 5 fractions; 2.4% 40 Gy in 8–10 fractions)	40.5% (30 Gy in 10 fractions)

Values are presented as percent or median (range), if not stated otherwise.

*SRS* stereotactic radiosurgery, *FSRT* fractionated stereotactic radiotherapy, *WBI* whole brain irradiation, *Gy* Gray, *RN* radionecrosis

### Concurrent administration of T-DM1 and SRS

Trastuzumab emtansine (T-DM1) holds a special position within HER‑2 targeted therapies as it is not only an antibody–drug conjugate but is also thought to potentially penetrate the blood–brain barrier more effectively, resulting in improved outcome in patients with brain metastases, as shown in the EMILIA trial. T‑DM1 combines the abilities of targeting HER2 receptors with trastuzumab and the cytotoxic abilities of the microtubule-inhibitory agent DM1 [31, 32].

Four of the included studies focused solely on concurrent use of T‑DM1, while two other studies additionally included the use of trastuzumab deruxtecan, another antibody–drug conjugate.

Stumpf et al. retrospectively examined 45 patients, of which 16 patients received T‑DM1 within a 4-week window of stereotactic radiation. In all, 10 patients (22%) developed clinically significant radionecrosis (CSRN), with a 13.5-fold (*p* = 0.02) increase in CSRN associated with receipt of T‑DM1 (39.1% vs 4.5%). Of 9 patients developing CSRN with T‑DM1, 6 patients received T‑DM1 concurrently. No further statistical analysis between the concurrent and sequential group was carried out. Logistic regression showed increasing numbers of SRS courses and age > 45 years as statistic risk factors for CSRN. Interestingly, the patient cohort consisted of patients with BCBM less than 45 years of age regardless of HER2 status and patients with HER2-positive BCBM independent of age. This selection could potentially limit the results, as presumable higher age in the HER2-positive group may influence the risk of radionecrosis, although age as a confounder was considered in the logistic regression. Despite this limitation, the authors did not only use a more clinically relevant outcome parameter (CSRN) but were also able to show molecular mechanisms behind radionecrosis, such as dysregulation of aquaporin 4 expression especially for T‑DM1 [20].

In 12 patients receiving T‑DM1—4 of them concurrently with SRS—the rate of radiation necrosis was higher in the concurrent group versus the sequential group (50% vs. 28.6%) according to a study by Geraud et al. This study defined concurrent administration as within a 1-month window but did not provide the used strategy for diagnosing radionecrosis or median follow-up and is limited by small sample size [25].

Mills et al. also described one case of radionecrosis in their study (3% rate of radionecrosis) that occurred after concurrent administration of T‑DM1. Sample size was 40 lesions (16 patients) total, of which 19 were treated concurrently. For this study the different definition of “concurrent” as within a 6-month window is important to take into consideration.

The heterogenous definition of concurrent use and consideration of timing of drug administration is also noticed when looking at the study by Id Said et al. Of 67 included patients with 223 lesions, 29 received T‑DM1, but this subgroup was only divided into administration prior to SRS (8 patients) and after SRS (21 patients, 14 of those within 1 year). Development of RN was observed in 18 patients total (27%), and T‑DM1 treatment post-SRS as well as radiotherapy dose were identified as independent risk factors. Median time from SRS to RN was 4.8 months; median follow-up was 15.6 months. Different categorization of timing reduces significance for our review, but identification of T‑DM1 as a risk factor must be acknowledged [21].

Lebow et al. additionally investigated the effect of a second HER2-targeted therapy, trastuzumab deruxtecan. Of 98 patients total, 35 patients were administered concurrently one of the two drugs. Radionecrosis was recorded as symptomatic radionecrosis (SRN); concurrent was defined as delivery within 7 days before or 21 days after SRS (approximately three half-lives). Considering a radiographic median follow-up of 12.4 months, concurrent administration of ADCs was associated with a higher risk for SRN (subdistribution hazard ratio [SHR] 4.01; 95% confidence interval [CI] 1.79–9.01; *p* < 0.001 [univariate analysis] and SHR 4.31; 95% CI 1.95–9.50; *p* < 0.001 [multivariate analysis]). This increased risk also applies for previously radiated lesions (42.0% vs. 9.4%) and grade 4 to 5 SRN (7.1% vs 0.7%) [26]. The significance of this study for our specific research question is compromised by the fact that mentioned statistical analyses were only carried out for all ADCs together, including a third substance, sacituzumab govitecan, which is not HER2-targeted. However, this affects only a small portion in the concurrent subgroup, as 7 of 42 patients received sacituzumab govitecan.

A second included study also investigated other ADCs. Koide et al. focused on impact of concurrent ADCs and radiotherapy on symptomatic radiation necrosis (SRN) in 168 patients. Used substances were trastuzumab emtansine (T-DM1) and trastuzumab deruxtecan (T-DXd). Concurrent administration took place in 19 of 63 HER2-positive patients and was defined as within 4 weeks before or after the first day of radiotherapy. Although this patient subgroup consisted of patients treated with different doses and fractionation of SRS (13 patients) as well as patients treated with WBRT (6 patients), the long median follow-up of 31 months and a multicenter study design must be recognized. Use of ADC in general—disregarding radiation differences—was associated with a significantly higher incidence of SRN (26.3% vs 8.7% total; 2‑year cumulative incidence 27.4% vs 7.0%, *p* = 0.014). Results did not include comparison of both substances as 44 of 48 patients received T‑DM1 and only 4 T-DXd [30].

### Concurrent administration of lapatinib and SRS

Lapatinib is a kinase inhibitor targeting HER2 receptors. It is administered orally and is typically prescribed in combination with capecitabine, trastuzumab or an aromatase inhibitor [33]. Two studies in this review investigated the concurrent use of lapatinib with stereotactic radiation.

Of 487 brain metastases in the study by Kim et al., 132 (27%) were treated concurrently. The 12-month cumulative incidences of radiation necrosis in the concurrent therapy group did not significantly differ from patients treated without concurrent lapatinib (1.0%, 95% CI 0.0–2.8% vs 3.5%, 95% CI 0.2–5.4%, *p* = 0.134), but risk factors for radiation necrosis such as larger lesion size could be confirmed. The control group in this study were patients not receiving lapatinib, but partly receiving other systemic therapies, which implies only a similar risk compared to other systemic therapies but not SRS alone. Furthermore, 87 of 132 lesions that were treated simultaneously with lapatinib, received additional trastuzumab during radiation, which was not accounted for in statistical analysis. Therefore, a potentially different influence of lapatinib compared to trastuzumab could be masked. Radiographic median follow-up in this study were only 8.2 months [34].

Parsai et al. included 126 patients with 47 lesions, of which 24 patients received lapatinib concurrent with SRS. The concurrent time window in this study was rather short with 5 days but was equivalent with five half-lives of the drug. The authors state that concurrent lapatinib was not associated with increased cumulative incidences of RN after 6, 12, and 24 months, but with increased lesion volume. Median radiographic follow-up was 17.1 months. In divergence to some other included studies, Parsai et al. analysed local failure and radiation necrosis on a per-patient basis instead of a per-lesion basis. Furthermore, 42 of 47 patients receiving lapatinib received an additional HER antibody [14].

### Concurrent administration of tucatinib and SRS

Tucatinib is a relatively new oral tyrosine kinase inhibitor targeting HER2 receptors. It was approved by the European Medicines Agency (EMA) in 2021 for use in patients who received at least two prior HER2− directed therapies [35]. The HER2CLIMB clinical trial not only showed prolongated survival but also reduced risk of intracranial progression and included a notably high percentage of patients with baseline brain metastases (46%) [36].

We could include one clinical study that investigated concurrent treatment with tucatinib and stereotactic radiosurgery for brain metastases in 22 patients with 135 lesions during 39 treatment sessions. Seventeen radiation sessions were delivered concurrently with tucatinib [27]. Khatri et al. report that 6 of 135 irradiated lesions developed symptomatic radiation necrosis after a median time of 9.5 months (range 7.4–20.73 months), but did not state whether those were in the concurrent or sequential therapy subgroup. While the authors themselves noted limitations due to retrospective design and small sample size, the relatively long median follow-up of this study with 20.8 months must be acknowledged, even if this median follow-up starts with first tucatinib administration. Furthermore, it is important to consider that daily oral treatment with tucatinib usually includes concurrent treatment with capecitabine and trastuzumab. Therefore, effects on various outcome parameters and especially risk of radionecrosis cannot be assigned to a singular substance.

### Basket studies on concurrent administration of various HER2-targeted therapies and SRS

Four included studies considered various HER2-directed therapies instead of a singular substance or substance group. This resulted in partly larger sample sizes, although effects sometimes could obviously not be attributed to singular substances in some studies.

Miller et al. included 547 patients with 3224 BM, of which a quite large subgroup (479 lesions) was HER2-positive. Within these, 109 (80%) received a HER2 antibody (mostly trastuzumab) concurrent with radiation therapy and 84 (38%) received a HER2/EGFR TKI (e.g. lapatinib) concurrently. Concurrent therapy was defined as within 5 biological half-lives for each drug. The authors reported a lower 12-month cumulative incidence of radiation necrosis for concurrent use of HER2/EGFR TKI with SRS (1.3% vs 6.3%; *p* = 0.001; HR: 0.23; 95% CI 0.07–0.78 [*p* = 0.02]), but no significant effect of concurrent HER2 antibodies on 12-month cumulative incidences (4.3% vs 5.8%; *p* = 0.30). Median radiographic follow-up was 10.1 months [13].

In a previous study with 1939 patients (but only 47 HER2-positive patients), Miller et al. analysed the concurrent use of HER2-targeted therapies on the risk for radiation necrosis and a window of 30 days around SRS was established for all substances. Even though HER2-positive histology doubled incidences of RN, the use of targeted therapies did not increase rates of RN significantly (5.9% vs 7.9%, *p* = 0.50 for HER2 antibodies and 0% vs 9%, *p* < 0.01 for lapatinib). Median radiographic follow-up was 12 months [28].

In this context, it is essential to highlight that the patients included in the two studies mentioned last, as well as the patient sample in the study by Parsai et al., most likely originate from the same hospital [13, 14, 28]. However, we still included all three publications as they partly analyse different aspects or slightly different cohorts.

Ippolito et al. [23] investigated the effect of trastuzumab combined with pertuzumab in 49 patients. Ten patients with 32 lesions received treatment within a 21-day window of SRS and one of them developed symptomatic radionecrosis. No further statistical analyses were carried out on this matter, but the authors stated that the larger lesion size in this patient could have influenced the occurrence of RN. Median follow-up in this study was comparatively long with 18.3 months.

A different, fixed-time outcome measure was used by Park et al. [29]. Radionecrosis after 12 months was assessed in 46 patients, of which 24 received various HER2 systemic treatment simultaneously (within a 4-week window) with SRS. This study observed that patients who were administered higher number of HER2-directed drugs during SRS, had a higher risk of developing radionecrosis (35.7% vs 5.6%, *p* = 0.047), but HER2-targeted therapy itself during SRS was not significant as a risk factor for development of RN (*p* = 0.59). In total, a rather large proportion with 16 of the 24 patients mentioned above developed RN. Patients not receiving systemic treatment in the same hospital where radiation took place (*n* = 68) were excluded in this study, as well as patients with death or loss to follow-up before the 12-month MRI. Especially the last exclusion criteria could distort results, as 12-month survival is not always reached in patients with advanced cancer [29].

## Discussion

This review highlights once more the partly conflicting research results on risk of radionecrosis after concurrent HER2 treatment with SRS.

While the two studies focusing on concurrent lapatinib could show no effect on risk for development of radionecrosis, the study focusing on tucatinib did not differ between sequential and concurrent use [14, 24, 27]. Concurrent administration of T‑DM1 or other ADCs seems to be associated with higher risk of radionecrosis as four of six included studies state significant effects, while the other two described cases of RN narratively [20–22, 25, 26]. Studies investigating various substances, mainly HER2 antibodies, mostly found no significant effect, although one study reported an even lower risk for RN with concurrent use of HER2/EGFR TKI (lapatinib) [13, 23, 28, 29].

Similar results are reported in other reviews, which also found possible increased radionecrosis risk for concurrent T‑DM1 but not HER2 inhibitors [37]. This heightened risk was also confirmed in two other meta-analyses [38, 39]. Khan et al. even reported a reduced risk for radionecrosis with administration of lapatinib in a systematic review and metanalysis with six studies. However, this review did not differentiate between SRS and whole brain irradiation and did not investigate effects of timing [40].

Furthermore, it is crucial to mention that although our review aimed to focus on a very specific substance group, a very heterogenous selection of studies was ultimately included, making them less comparable. Variations ranged from different sample sizes to varying definitions of concurrent use and different outcome parameters regarding radionecrosis. Median follow-up ranged from 8.2 to 31 months. Length of follow-up holds particular significance in investigating RN, as typical RN occurs 6–18 months after RT but also much later as “late RN” or “ultra-late RN”. Although follow-up can be limited due to death from extracranial progression or other reasons, striving for follow-ups as long as possible, increases value of clinical studies regarding RN [19]. Other aspects contributing to heterogeneity have emerged from differences in radiation parameters as presented in Table 2. What all included studies have in common is a partly limiting retrospective study design.

When statistically evaluating substance induced effects on occurrence of radiation necrosis as a study endpoint, a variety of confounding factors must be considered, as patient and radiation characteristics may influence occurrence rates [2, 11, 12]. This can be achieved through utilization of different statistical methods such as regression analyses. As mentioned in the results part of this review, mostly competing risk regression analysis, cumulative incidence analysis, logistic regression, and Gray’s test were performed. However, the included studies lack comparability in terms of how confounders were statistically addressed. In general, covariates often seem to be chosen considering “previously identified risk factors for RN, variables prognostic for survival, and biological variables” as Miller et al. stated [28]. For instance, Id Said et al. utilized age, RT dose, lesion volume, intracranial location, number of BM and prior whole brain RT as covariates in their competing risk regression analysis [21]. Miller et al. additionally considered sex, extracranial metastases, disease histology, mutational status, treatment with systemic therapies, prior surgery, conformality index, heterogeneity index, lesion laterality and maximum diameter as possible confounders [13, 28]. Other publications included did not disclose confounders or methods in detail or were unable to account for confounders due to smaller sample size and therefore not meeting requirements for such analyses. These include for example publications from Geraud et al. (smaller sample size with descriptive statistics), Lebow et al., Mills et al., Park et al. and Ippolito et al. [22, 23, 25, 26, 29].

Additionally, this research topic depicts not the only existing knowledge gap as definite diagnosis of radionecrosis represents a challenge to this day. Diagnosis of radiation necrosis can be difficult to achieve as there is no sole method of diagnostic imaging with high enough specificity to differentiate between local recurrence and radiation necrosis with sufficient reliability and frequency. Indicative options for diagnostic imaging include magnetic resonance imaging (MRI), perfusion MRI and 18F-fluorethylthyrosine-positron emission tomography (FET-PET), but histology remains the gold standard for definite diagnosis [17, 18].

Moreover, an association with increased risk of radionecrosis has also been shown for other targeted therapies when administered concurrently. This includes BRAF/MEK inhibitors, epidermal growth factor receptor (EGFR)-TKIs and vascular endothelial growth factor receptor (VEGFR)-TKIs [16, 41].

Limitations of this review include that only databases PubMed/Medline and Cochrane library were searched and that most steps in search, eligibility testing and data extraction were only performed by one reviewer due to practical/organizational reasons.

As clinical implications still appear difficult to claim due to conflicting results and various limitations, more research on the specific matter of this review is unquestionably needed. Related to this, it appears reasonable to strive for a standardization of terminology, particularly the definition of “concurrent use”, for better comparability of research results. Despite the findings of Stumpf et al. which provided indications on the pathomechanisms of radionecrosis by demonstrating dysregulation of aquaporin 4 channels, the underlying mechanisms remain mostly unclear and require more research as well. Other factors like VEGF expression, hypoxia pathways and disruption of blood–brain barrier also seem to play a role [19, 42, 43].

## Conclusion

The concurrent use of trastuzumab emtansine (T-DM1) and other antibody–drug conjugates (ADCs) appears to be linked with a heightened risk for development of radionecrosis. Risk for radionecrosis (RN) associated with other HER2-targeted therapies remains mostly unclear. When evaluating included studies, it is crucial to consider the heterogenous study selection with different used substances and varying definitions of concurrent use and radionecrosis as well as different outcome parameters. Additionally, included studies are partly limited by sample size and retrospective study designs. As a result, clinical implications remain challenging to establish, highlighting the need for further research on this topic.

## Data Availability

Detailed data and full search strategy are available upon request to the corresponding authors.
